# A New De-Noising Method Based on Enhanced Time-Frequency Manifold and Kurtosis-Wavelet Dictionary for Rolling Bearing Fault Vibration Signal

**DOI:** 10.3390/s22166108

**Published:** 2022-08-16

**Authors:** Qingbin Tong, Ziyu Liu, Feiyu Lu, Ziwei Feng, Qingzhu Wan

**Affiliations:** 1 School of Electrical Engineering, Beijing Jiaotong University, Beijing 100044, China; 2 Beijing Rail Transit Electrical Engineering Technology Research Center, Beijing 100044, China; 3 School of Electrical and Control Engineering, North China University of Technology, Beijing 100144, China

**Keywords:** signal de-noising, phase space reconstruction, time-frequency manifold, Renyi entropy, sparse representation

## Abstract

The transient pulses caused by local faults of rolling bearings are an important measurement information for fault diagnosis. However, extracting transient pulses from complex nonstationary vibration signals with a large amount of background noise is challenging, especially in the early stage. To improve the anti-noise ability and detect incipient faults, a novel signal de-noising method based on enhanced time-frequency manifold (ETFM) and kurtosis-wavelet dictionary is proposed. First, to mine the high-dimensional features, the C-C method and Cao’s method are combined to determine the embedding dimension and delay time of phase space reconstruction. Second, the input parameters of the liner local tangent space arrangement (LLTSA) algorithm are determined by the grid search method based on Renyi entropy, and the dimension is reduced by manifold learning to obtain the ETFM with the highest time-frequency aggregation. Finally, a kurtosis-wavelet dictionary is constructed for selecting the best atom and eliminating the noise and reconstruct the defective signal. Actual simulations showed that the proposed method is more effective in noise suppression than traditional algorithms and that it can accurately reproduce the amplitude and phase information of the raw signal.

## 1. Introduction

As an important part of rotating machinery, the dynamic performance of rolling bearing affects the safe and stable operation of rotating machinery. If the defects cannot be found in time, the mechanical equipment will stop running, or major safety accidents will be caused [1,2,3]. Therefore, the rolling bearing failure is the root cause of rotating machinery failure, which must be found as soon as possible to avoid the economic losses and disasters.

As the important information of fault diagnosis, the periodic transient shock contained in the bearing vibration data reflects the important dynamic information of the fault bearing. However, the measured vibration signal is often complex, non-stationary, and contains a large amount of background noise. The useful fault information is often weak and difficult to identify. At present, the commonly used signal de-noising methods include the wavelet denoising method, modal decomposition method, spectrum subtraction method, and so on. Qiu et al. [4] successfully extracted weak pulses from rolling bearing fault signals by using Morlet wavelet. Dron et al. [5] used spectral subtraction to suppress time invariant noise and improve signal kurtosis and peak factor. Abdelkader et al. [6] optimized empirical mode decomposition through the threshold method for early faults identification of rolling bearings. Zhang et al. [7] utilized the variational modal decomposition algorithm to extract fault information of gears and rolling element bearing. However, the traditional methods have their own shortcomings, and the wavelet de-noising method is difficult to deal with in terms of the white noise widely existing in each frequency band. Although the spectral subtraction method has a good suppression effect on steady-state noise, it is not suitable for dealing with background noise in nonlinear and non-stationary vibration signals. Mode decomposition has the problems of endpoint effect and mode aliasing.

Chaos theory is an important part of nonlinear science. It reflects a “random” state of motion and reveals the internal order information in deterministic nonlinear systems [8]. Takens introduced chaos theory into nonlinear time series analysis [9]. He believes that the attractor of the chaotic system can be reconstructed from the measured single-variable time series, and the judgment, analysis, and prediction of the chaotic system can be carried out according to the properties of the attractor. The phase space reconstruction that reflects the dynamic characteristics of the system needs a long enough time series and a reasonable choice of reconstruction parameters, embedding dimension and delay [10]. There are mainly two views on the selection of embedding dimension and time delay: The first view is that the two are unrelated, representing algorithms such as Cao’s method [11]. Zhang et al. [12] used mutual information algorithm and Cao’s method to determine the appropriate delay time and the optimal embedding dimension and effectively identified the chaotic signals. The second view holds that the embedding dimension and the time delay are correlated, representing the C-C method [13], the time window method [14]. Wang et al. [15] used the C-C method to process the raw wind speed sequence and input the reconstructed phase space into the subsequent model to successfully predict wind speed. Specifically, the C-C method does not consider the effect of the sequence length of a one-dimensional vibration signal on the performance of the algorithm and its robustness is poor. Cao’s method needs a time delay to calculate the size of the embedded dimension. On this basis, this paper, on the basis of exploring the principles of C-C method and Cao’s method, proposes to use C-C algorithm and Cao’s method to jointly determine the time delay and embedding dimension, and map vibration signals into high-dimensional space to mine their essential features.

Manifold learning can effectively mine the embedded coordinates of low-dimensional manifolds in reconstructed high-dimensional space and realize the learning and enhancement of data features and essential information [16,17,18,19,20]. On the basis of the LTSA algorithm, the LLTSA [21] algorithm does the work of local linear extension, avoids the loss of many sensitive features, and is more suitable for the extraction of nonlinear features in rolling bearing fault diagnosis. Tang et al. [22] used the LLTSA algorithm to compress the high-dimensional vectors of the samples so that the samples have better resolution. In recent years, many scholars began to combine time-frequency analysis with manifold learning, which not only analyzed the time-frequency manifold structure of signals but also achieved a good effect of noise suppression [23,24,25]. He et al. [26] analyzed the nonlinear time-frequency manifold structure of defective signals, and the extracted signal features were suitable for the diagnosis of mechanical faults. Li et al. [27] extracted the time-frequency manifold of RF signals and successfully separated and classified the signals. There are many optimization methods for input parameters of the LLTSA algorithm; Kumar et al. [28] used the frequency factor to optimize the optimal neighborhood. However, the applicability of these methods to time-frequency manifold scenarios is not high, so this paper proposes a gridded parameter search method based on Renyi entropy to optimize LLTSA. Finally, the low-dimensional manifold with the highest time-frequency aggregation is obtained, which is called the enhanced time-frequency manifold (ETFM). Because the computation process of LLTSA algorithm is nonlinear, ETFM inevitably loses the amplitude information of transient impacts which we are concerned about.

The purpose of sparse representation is to represent most or all of the information of the signal with fewer atoms, which can complete the detection of the internal structure of the data while maintaining the transient amplitude of the signal [29,30]. He et al. [31,32] combined manifold learning with sparse representation and proposed time-frequency manifold sparse algorithm, which achieved good results in fault diagnosis of rotating machinery. Tang et al. [33] used sparse representation to extract fault features submerged in noise. The dictionary determines the error between the sparse representation result and the raw signal, which has great influence on the final fault feature extraction and signal de-noising. At present, the dictionary can be divided into analysis dictionary and learned dictionary. Analysis dictionary uses fixed basis function to construct a dictionary, which has the advantage of being fast and concise. Zheng et al. [34] introduced the Gabor multi-channel model to construct the Gabor dictionary and proved the usability of this method in the field of face recognition. Fan et al. [35] used the Morlet dictionary for de-noising and feature extraction of gearbox fault signals. In different application scenarios, the adaptability of analysis dictionary is poor, while the adaptability of learned dictionary is strong because it is learned from data. Zhou et al. [36] used shift-invariant dictionary learning to extract fault shock of mechanical equipment signals. Ren et al. [37] used data sets with known fault types to train the dictionary, which improved the accuracy of fault diagnosis. Kong et al. [38] proposed a discriminative dictionary-learning-based sparse representation classification framework for intelligent planet-bearing fault identification. The quality of the sparse representation results obtained by learned dictionary largely depends on the dictionary learning algorithms, which are complex in computation and tedious in the optimization process. Therefore, combining the advantages of analysis dictionary and learned dictionary, this paper proposes the construction of kurtosis-wavelet dictionary.

In order to reduce the distortion degree of reconstructed phase space, improve the noise suppression effect of time-frequency manifold learning, and highlight the feature extraction ability of sparse representation, we propose a new de-noising method based on ETFM and kurtosis-wavelet dictionary for rolling Bearing Fault vibration signal. The denoised signal obtained by this method has a very high SNR and retains the amplitude information of the raw signal, which lays a foundation for fault feature extraction. The main contributions of this paper are as follows:

1. We propose a method of using C-C method and Cao’s method jointly to determine the time delay and embedding dimension of phase space reconstruction, which reduces the distortion degree of reconstruction space and highlights the fault characteristics.
2. LLTSA is optimized by a gridded parameter search method based on Renyi entropy to obtain ETFM with high time-frequency resolution.
3. The proposed kurtosis-wavelet dictionary can adaptively select the optimal atomic position with the change of kurtosis, which improves the noise suppression effect and feature extraction ability of sparse representation.

The remaining parts of this paper are organized as follows: Section 2 briefly introduces the principles of our proposed innovative approach, and Section 3 shows the complete framework of the signal de-noising method proposed by this paper. Section 4 presents and discusses the experimental results. In Section 5, ablation and comparison experiments are designed on the basis of the innovative work in this paper. Section 6 summarizes the work of this paper.

## 2. Methodology

### 2.1. Parameter Optimization Phase Space Reconstruction

Phase space reconstruction (PSR) is an important tool to mining high-dimensional features of signals, and it is even more indispensable to reveal the dynamics law and the state space attribute of observation data. At present, the construction of phase space is essentially based on the time delay method of Taken’s theorem, which holds that the evolution of any component in the system is determined by other components interacting with it. The theorem shows that the attractor of chaotic system can be reconstructed from the measured single variable time series. Therefore, the information of these relevant components is implicit in the development process of any component. To reconstruct the phase space of the system, only one component $x=\left[x_{1},x_{2},\cdots ,x_{n}\right]$ needs to be investigated, and the m-dimensional phase space vector can be found through the observations at different time delay points; the specific formula is as follows:

(1) $$X_{i}^{m}=\left[x_{i},x_{i+\tau },\cdots ,x_{i+(m-1)\tau }\right]$$

where m represents the dimension of the phase space, i represents the signal point, τ represents the time delay, and m represents the embedding dimension. N=n−(m−1)τ, as long as N≥2d+1 (d is the dimension of the chaotic attractor), wherein an equivalent phase space can be reconstructed. In this phase space, the raw dynamic system can be restored, and the properties of its attractor can be studied to judge, analyze, and predict the chaotic time series. According to the time series $\left\{X_{i}^{m}|i=1,2,\cdots ,N\right\}$, these N vectors are aligned to obtain the timing matrix expressed as $P\in R^{m\times N}$, and the corresponding relationship between any element in the phase space and the initial one-dimensional time series signal is expressed by Equation (2).

(2) $$P_{\left(j,k\right)}=x_{k+(j-1)\tau },j\in [1,m],k\in [1,N]$$

In order to construct the phase space from the time series, the parameter m and the appropriate sampling interval τ are very important. In theory, the selection of τ can be almost arbitrary. However, in the actual system, τ should also be determined by repeated trial and error. If τ is too small, the orbit of phase space tends to a straight line; if τ is too large, the data points will be concentrated in a small area of the phase space.

Cao’s method only needs time delay τ and a small amount of data to calculate the embedding dimension m. The C-C method can calculate τ and $\tau_{w}$ simultaneously through correlation integral, but it has a certain selectivity for the length of time series, which is not suitable for signal with short length. Moreover, the calculation results of τ and $\tau_{w}$ by the C-C method are unstable. Therefore, we propose to jointly determine τ and m by the C-C method and Cao’s method. The specific steps are as follows:

1. Determine the optimal time delay τ by the C-C method [13]

Given a time series of length N $x=\left\{x_{n}|n=1,2,\cdots ,N\right\}$, with delay *t* and embedding dimension *k*; reconstruct the phase space $X_{i}\left(n\right)=\left\{x_{i}\left(n\right),x_{i}\left(n+t\right),x_{i}\left[n+\left(m-1\right)t\right]\right\}$, where *i* = 1, 2, …, *M*, $X_{i}\left(n\right)$ is a point in the phase space, which is used to represent the association of embedded timing signals The points are

(3) $$C\left(k,N,r,t\right)=\frac{2}{M\left(M-1\right)}\sum_{1\leq i<j\leq M}\theta \left(r-d_{ij}\right)$$

where $d_{ij}=\|X_{i}-X_{j}\|$, r is the size of the neighborhood radius; if x<0, θ(x)>0; and if x<0, θ(x)>0. The time series $x=\left\{x_{n}|n=1,2,\cdots ,N\right\}$ is decomposed into t subsequences. If the length of each subsequence is the same and the two subsequences do not overlap each other, t is the reconstruction delay, and N represents the integer multiple of t, i.e.,

(4) $$\begin{array}{c} x^{1}=\left\{x_{n}|n=1,t+1,\cdots ,N-t+1\right\}\\ x^{2}=\left\{x_{n}|n=2,t+2,\cdots ,N-t+2\right\}\\ \vdots \\ x^{t}=\left\{x_{n}|n=t,2t,\cdots ,N\right\} \end{array}$$

The statistics defined in the above analysis are calculated by using the block average strategy:

(5) $$S_{1}\left(k,N,r,t\right)=\frac{1}{t}\sum_{s=1}^{t}\left[C_{s}(k,N/t,r,t)-C_{s}^{k}(1,N/t,r,t)\right]$$

Let N→∞.

(6) $$S_{2}\left(k,r,t\right)=\frac{1}{t}\sum_{s=1}^{t}\left[C_{s}(k,r,t)-C_{s}^{k}(1,r,t)\right]$$

Due to the limited length of the actual sequence, the obtained $S_{2}\left(k,r,t\right)$ is generally not zero, and $S_{2}\left(k,r,t\right)\sim t$ reflects the autocorrelation of the time series. According to the autocorrelation principle of the value of *τ*, the first zero point of $S_{2}\left(k,r,t\right)\sim t$ is selected as the optimal delay τ, which is defined as

(7) $$\Delta S_{2}\left(k,t\right)=\max\left\{S_{2}\left(k,r_{j},t\right)\right\}-\min\left\{S_{2}\left(k,r_{j},t\right)\right\}$$

$\Delta S_{2}\left(k,t\right)$ finally is obtained the maximum deviation result of $S_{2}\left(k,r,t\right)\sim t$ for all neighborhood radius r. From the above analysis, it can be concluded that the first value of $S_{2}\left(k,r,t\right)\sim t$ zero point or the first local minimum point of $\Delta S_{2}\left(k,t\right)\sim t$ can be used as the optimal delay τ.

2. Determine the optimal embedding dimension m [11]

Construct an m-dimensional phase space,

(8) $$a_{\left(i,m\right)}=\frac{\left\|x_{i}(m+1)-x_{n(i,m)}(m+1)\right\|}{\left\|x_{i}(m)-x_{n(i,m)}(m)\right\|},\;i=1,2,\cdots ,N-m\tau$$

where ‖·‖ represents the norm of the vector; $X_{i}\left(m\right)$ and $X_{n\left(i,m\right)}\left(m\right)$ represent the data point of the i-th vector and the nearest neighbor vector in the m-dimensional phase space, respectively; $X_{i}\left(m+1\right)$ and $X_{n\left(i,m\right)}\left(m+1\right)$ represent the *i*-th vector of the (m+1)-dimensional phase space and its nearest point, respectively; and n(i,m) is greater than 1 and less than or equal to N−mτ. If $X_{n\left(i,m\right)}\left(m\right)$ is equal to $X_{i}\left(m\right)$ during the calculation, the next nearest vector needs to be found according to the definition of norm. The norm is defined as

(9) $$E\left(m\right)=\frac{1}{N-m\tau }\sum_{i=1}^{N-m\tau }a(i,m)$$

(10) $$E_{1}(m)=\frac{E(m+1)}{E(m)}$$

Due to the different natures of time series, it is difficult to judge whether $E_{1}\left(m\right)$ tends to be stable, so a judgment criterion is added:

(11) $$E^{+}\left(m\right)=\frac{1}{N-m\tau }\sum_{i=1}^{N-m\tau }\left|X(i+m\tau )-X[n(i,m)+m\tau ]\right|$$

(12) $$E_{2}(m)=\frac{E^{+}(m+1)}{E^{+}(m)}$$

**Figure 1:**
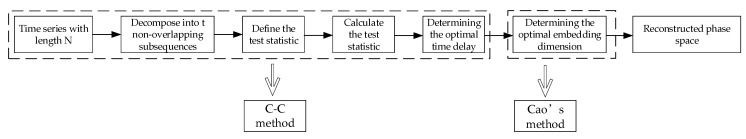
PSR parameter selection process.

To express the method more intuitively and simply for optimizing the selection of phase space reconstruction parameters proposed in this paper, the process is shown in Figure 1.

### 2.2. Enhanced Time-Frequency Manifold

Time-frequency manifold (TFM) is an inherent nonlinear manifold structure that is described on the time-frequency distribution. Through the time-frequency analysis of the reconstruction space, time-frequency manifold learning is used to mine the features of the signal embedded in the low-dimensional space from the high-dimensional space. Since the non-stationary and nonlinear characteristics of the signal itself are analyzed in the learning process, time-frequency manifold learning can effectively mine and enhance the time-frequency modes of the signal and describe the time-frequency distribution.

According to the PSR introduced in Section 2.1, the time series matrix $P\in R^{m\times N}$ is obtained. Then, we need to mine the fault feature information through the potential manifold in the high dimension. This paper uses the short-time Fourier transform (STFT) algorithm to perform time-frequency analysis on m time series $P_{j}$ in the matrix P to obtain complex matrix $S_{j}$. To maintain the phase characteristics of the raw signal and ensure the consistency of the reconstructed signal and the raw signal, $S_{j}$ is divided into an amplitude matrix $A_{j}$ and a phase matrix $\theta_{j}$. The magnitude matrix $A_{j}$ obtained from each time series $P_{j}$ is arranged to form a time-frequency manifold in a high-dimensional space.

**Figure 2:**
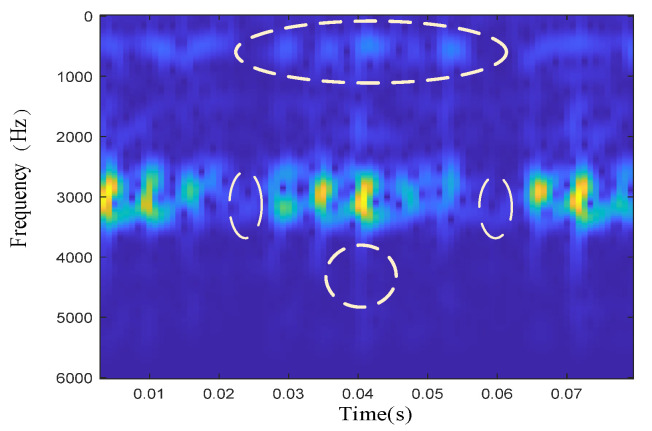
Time-frequency diagram of the inner-race defective signal.

The raw signal is not stable, so the time-frequency manifold must be mixed with quite a lot of noise. Figure 2 shows the results of the time-frequency manifold of the inner-race defective signal. It can be clearly seen that at approximately 3000 Hz, the defective signal forms a resonance band. However, from the positions marked with dotted lines in the figure, it can be seen that a large amount of noise is mixed between the 3000 Hz resonance band and other frequency bands. To extract the defective features submerged in the noise, this subsection introduces an enhanced manifold learning algorithm to mine the low-dimensional nonlinear time-frequency manifold structure embedded in the high-dimensional space.

The LLTSA algorithm is a typical nonlinear manifold learning algorithm. This algorithm approximates the local part of the raw signal through the limit of the tangent space and uses the local tangent space arrangement to map the signal in the low-dimensional space. LLTSA is a partial maximal linear expansion of the approximate linear tangent space based on the LTSA algorithm. It includes the characteristics of principal component analysis (PCA) and LTSA. While extracting the nonlinear features of high-dimensional data, local neighborhood information is preserved. Compared with algorithms such as LTSA, it avoids the loss of many sensitive features and is suitable for dimension reduction of nonlinear features.

The two input parameters of the LLTSA algorithm are the dimensionality reduction dimension *d* and the neighborhood parameter *k*. The specific value of the parameter is very important to the final noise suppression effect. If *d* is too large, the low-dimensional manifold will contain too much redundant information, and the noise suppression effect will be poor; if *d* is too small, the low-dimensional manifold will lose some information and miss the defective features in the manifold structure. If *k* is too large, it will affect the division of the linear block range of the algorithm; if *k* is too small, it will reduce the correlation of the neighborhood structure. For LLTSA, if the constant parameters are selected blindly, it cannot adapt to all application scenarios, which will adversely affect signal de-noising. In this paper, a method based on Renyi entropy is proposed to optimize the input parameters of LLTSA. Renyi entropy is a kind of information entropy that is an objective index to evaluate energy concentration. The smaller the value is, the higher the energy concentration. The specific calculation formula of Renyi entropy is

(13) $$H_{\alpha }(x)=\frac{1}{1-\alpha }log({\sum_{i=1}^{n}p(i)}^{\alpha })$$

We take the Renyi entropy value of TFM obtained after LLTSA dimensionality reduction as the grid search target. Take the (d,k) point corresponding to the smallest Renyi entropy value as the optimal input of LLTSA. The specific steps of LLTSA parameters optimization based on Renyi entropy are as follows:

If the raw signal $x=\left[x_{1},x_{2},\cdots ,x_{n}\right]$, then

1. The time delay *τ* and the embedding dimension m are obtained by the C-C and Cao’s methods, and the phase space matrix $R^{m\times N}$ is obtained by the PSR.
2. The time-frequency analysis is performed on each time series $P_{j}$ of the phase space matrix $R^{m\times N}$ by the STFT, and then the amplitude matrix $A_{j}$ is obtained.
3. Set the grid search initialization parameters of LLTSA, the search step size is 1
4. Using the grid search method, the Renyi entropy value of TFM in each search is calculated, and the (d,k) corresponding to the smallest Renyi entropy value is taken as the optimal input parameters of LLTSA.

After optimizing LLTSA by grid search, we obtain low dimensional time-frequency structure enhanced time-frequency manifold (ETFM). Compared with the TFM obtained without optimization, ETFM has the highest time-frequency aggregation and the best noise suppression effect. In the next subsection, we use sparse representation algorithm to reconstruct ETFM.

### 2.3. Sparse Representation Based on Kurtosis-Wavelet Dictionary

In Section 2.2, we obtained ETFM with a good noise suppression effect through time-frequency manifold learning, but because of the nonlinear calculation in dimension reduction, ETFM loses the amplitude information of the raw signal. In this subsection, we use a sparse representation algorithm to overcome the distortion of ETFM. As the key of sparse representation, dictionary has an important influence on the final result of sparse representation. Therefore, kurtosis-wavelet dictionary is proposed in this paper. The kurtosis-wavelet dictionary not only has a high matching degree with the transient pulses, but also avoids the residual noise in ETFM to the greatest extent. Through the sparse representation of ETFM, the final result not only reproduces the amplitude information of the raw signal, but also has the advantage of low SNR of ETFM.

#### 2.3.1. Sparse Representation Principle

The sparse analysis of the signal refers to the overcomplete representation of the signal on the redundant dictionary. For signal $x\in H^{L}$, there is a redundant dictionary $D=\left\{\;d_{i},i=1,2,\ldots ,K,\;\|d_{i}\|=1\right\}$; then, the sparse expression of signal x on dictionary D is

(14) $$x=\sum_{m=1}^{M}g_{km}d_{km}$$

km is the selected atom label in the dictionary, and $g_{km}$ is the coefficient of the corresponding atom $d_{km}$. The sparsity of the signal is M (M≪K), which is the number of nonzero coefficients.

Because the result of sparse representation is not unique, it is necessary to solve the optimal solution of the sparse representation problem in order to achieve the best representation of the signal. According to Equation (14), the optimal solution must be the sparsest solution, that is, the solution with the least non-zero value in the coefficient. The greedy algorithm is a commonly used sparse representation algorithm. Its specific principle is to use the least square method to carry out local optimization according to the current data and obtain the final result through repeated iteration. The orthogonal matching pursuit (OMP) algorithm, as a typical greedy algorithm, is improved on the traditional matching pursuit (MP) algorithm. By using the Gram–Schmidt process to orthogonalize the projection direction, the approximate representation of the signal is continuously improved in this new way. The main idea is to project the signal into a redundant dictionary in each iteration so that the approximation error is minimized. Because the dictionary atoms filtered each time can be orthogonal, the OMP algorithm has a faster convergence rate than the MP algorithm. This paper uses the OMP algorithm in the process of solving the sparse expression.

#### 2.3.2. ETFM Reconstruction Using Kurtosis-Wavelet Dictionary

In the process of solving the sparse representation of the signal, the atoms in the dictionary will inevitably match with the noise in the signal. This would bring useless information to the final sparse representation result, which will affect the extraction effect of signal time-frequency features. This section proposes a method to construct a kurtosis-wavelet dictionary to address this problem.

First, according to the solution principle of sparse representation, the atoms closest to the raw signal must be screened in each iteration process, so the similarity between the transient pulse atoms in the dictionary and the signal fault impacts greatly affects the effect of signal sparse expression. Here, the Morlet wavelet matching periodic transient shocks are chosen to construct one-dimensional time-domain impulse atoms. Its time domain formula is as follows:

(15) $$\psi (t;\tau ,f,\xi )=\{\begin{array}{c} e^{-\xi /\sqrt{1-\xi^{2}}{\left[2\pi f(t-\tau )\right]}^{2}}cos(2\pi f(t-\tau )),\left|t-\tau \right|\leq W\\ 0\;\;\;\;\;\;\;\;\;\;\;\;\;\;\;\;\;\;\;\;\;\;\;\;\;\;\;else \end{array}$$

In the formula, the single impact duration W and parameters τ, f, and ξ determine the wavelet waveform and characteristics. To meet the analysis requirements in the time-frequency domain in this paper, STFT is used to convert one-dimensional atoms into two-dimensional atoms, and the calculation process of constructing the time-frequency dictionary $D_{tf}$ is as follows:

(16) ψ(k,v,kτ,f,ξ)=ST(ψ(t,τ,f,ξ))

ST represents the STFT of atoms in the time domain; k and v represent the time and frequency in the time-frequency domain, respectively; and kτ is the time index of the wavelet pulse.

In addition, this paper proposes a method based on kurtosis to determine the specific position of atoms in the time domain, so that the atoms can avoid the noise in the signal as much as possible and match the transient impulses. The kurtosis is a dimensionless index parameter that is very sensitive to the impulse in time series and is especially suitable for the judgment of the defective signal of the rolling bearing. Its calculation formula is

(17) $$K=\frac{E{\left(X-\mu \right)}^{4}}{\sigma^{4}}$$

where μ represents the mean value of the time series signal, σ represents the standard deviation of the time series signal, and E(⋅) is the expected value obtained. The larger the kurtosis value is, the more impulse components in the signal. To distinguish the impacts submerged in the noise, this study draws on the idea of window shifting in the STFT. Taking the raw signal of 1000 points as an example, we set the window length to 50, and then the non-overlapping moving window intercepted the signal, finally obtaining a total of 20 signals. Additionally, the kurtosis value of each intercepted signal was calculated and sorted. According to the definition and characteristics of kurtosis, there is a high probability of defective impacts at the location of the signal segments with large kurtosis values. Therefore, we chose to build atoms only at the positions corresponding to the signal segments with larger kurtosis values and combine these atoms to complete the construction of the kurtosis-wavelet dictionary.

**Figure 3:**
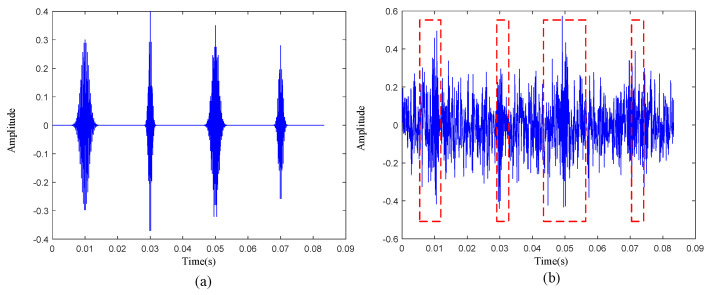
(**a**) Simulated signal without noise. (**b**) Simulated signal with a signal-to-noise ratio of −6 dB.

To further illustrate the specific steps of the dictionary construction method proposed in this paper and prove its effectiveness, a set of simulation signals are introduced here. The time-domain signal is shown in Figure 3a. To test the universality of this method, four different defective impacts are simulated here. The specific formula is

(18) $$s=\sum_{i=1}^{4}A_{i}\psi_{i}$$

where $\psi_{i}\left(t;\tau_{i},f_{i},\xi_{i}\right)=\{\begin{array}{c} e^{-\xi_{i}/\sqrt{1-\xi_{i}{}^{2}}{\left[2\pi f_{i}\left(t-\tau \right)\right]}^{2}}cos\left(2\pi f_{i}\left(t-\tau_{i}\right)\right),\left|t-\tau_{i}\right|\leq W\\ \;0\;\;\;\;\;\;\;\;\;\;\;\;\;\;\;\;else \end{array}$, $A_{i}=\left[0.3,0.4,0.35,0.28\right]$,$\;f_{i}=\left[3000,3200,3600,3300\right]$, $\tau_{i}=\left[0.01,0.03,0.05,0.07\right]$, and $\xi_{i}=\left[0.001,0.005,0.002,0.003\right]$.

To restore the defective signal in the real scene, white noise with a signal-to-noise ratio of −6 dB was added to the simulation signal, and the time-domain waveform is shown in Figure 3b. According to the construction method of kurtosis-wavelet dictionary, we first set the window length to 50, moved the window without overlapping in the time sequence, and calculated the kurtosis value of each intercepted signal. The average kurtosis value of the 20-segment signal was 2.8476, and the specific positions of the 8-segment signals with kurtosis greater than the average kurtosis value are shown in the red dotted box in Figure 3b. We chose to set atoms at these positions. According to the comparison of Figure 3a,b, it can be seen that these atoms were placed in the positions of the transient pulses, which avoids noise.

To make full use of the noise suppression effect of the ETFM, the transient pulses on the ETFM is learned to realize the mining of defective impact characteristics. In this paper, the time-frequency image of ETFM is used as the training sample of the kurtosis-wavelet dictionary $D_{tf}$, and the time-frequency atoms that best match the transient characteristics of ETFM are selected by the OMP algorithm. These time-frequency atoms are combined into a new dictionary $D_{tf1}$. Then, $D_{tf1}$ is used to sparsely represent m magnitude matrices $A_{j}$ of the raw signal to obtain $\hat{A_{j}}$. The specific formula is as follows:

(19) $$c_{M,j}={\left({D_{tf1}}^{T}D_{tf1}\right)}^{-1}{D_{tf1}}^{T}A_{j}$$

(20) $$\hat{A_{j}}=D_{tf1}c_{M,j}$$

Finally, the m reconstructed $\hat{A_{j}}$ and m $\theta_{j}$ are combined, and the reconstructed time domain signal can be obtained through the inverse STFT and PSR synthesis technology.

## 3. Complete Framework of the Proposed Method

**Figure 4:**
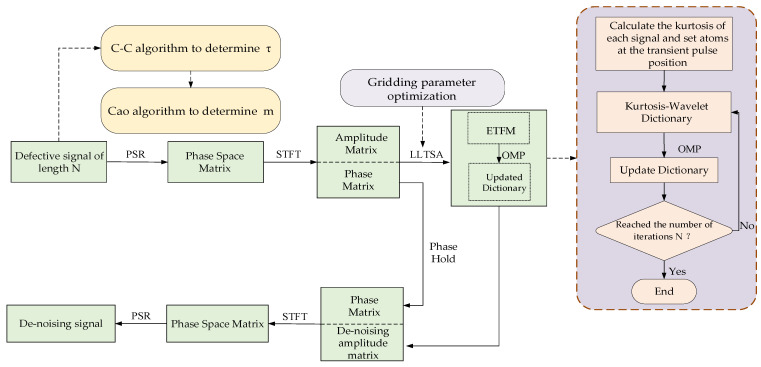
Flow chart of the signal reconstruction algorithm.

The complete framework is shown in Figure 4. The specific steps are as follows:

1. PSR: the C-C method and Cao’s method jointly determine the best time delay τ and the best embedding dimension m, and the PSR technology maps the raw signal to the high-dimensional space. High-dimensional spatial time-frequency features are mined by STFT and divided into amplitude matrix and phase matrix.
2. Enhance time-frequency manifold learning: LLTSA is optimized by the gridding search method, and the important features in the high-dimensional space are mined to obtain ETFM, which completes the preliminary noise reduction of the signal.
3. Kurtosis-wavelet dictionary generation: divide the raw signal into several segments and calculate the kurtosis of each segment. Set time-frequency wavelet atoms in the signal segment with large kurtosis to complete the construction of kurtosis-wavelet dictionary
4. Sparse representation: use the OMP algorithm to solve the sparse representation problem of ETFM and update the kurtosis-wavelet dictionary at the same time. The reconstruction result of the amplitude matrix is obtained by Equations (19) and (20). Then, the reconstructed amplitude matrix is combined with the phase matrix and restored to a one-dimensional signal by the inverse STFT and phase space reconstruction technology.

## 4. Experimental Results

In this section, the signal de-noising method proposed in this paper is experimentally verified by the measured defective signals of the inner-race, outer-race, and rolling-element of the rolling bearing. The measured vibration signal of the rolling bearing used in the analysis came from the test data of the bearing test bench built by the Electrical Engineering Laboratory of Case Western Reserve University in the United States.

**Figure 5:**
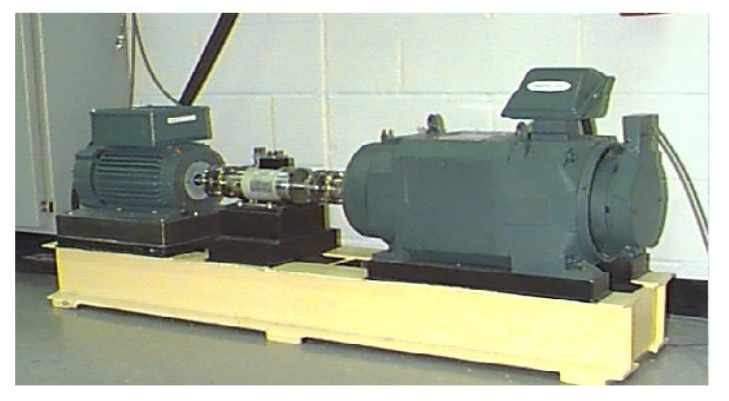
Bearing test platform.

Figure 5 shows the bearing test bench. The test bearing supports the main shaft of the motor. The model of the test bearing is 6205-2RS JEM SKF. Under the bearing corresponding to the bearing seat at the driving end of the motor spindle, also known as the bearing load area, an acceleration sensor was installed to test the vibration change signal of the bearing. The sampling frequency of the test bench was 12K. The defect-related parameters of three signals to be analyzed are listed in Table 1. 

### 4.1. Experimental Results of Inner-Race Defective Signal

#### 4.1.1. Experimental Results of PSR

**Figure 6:**
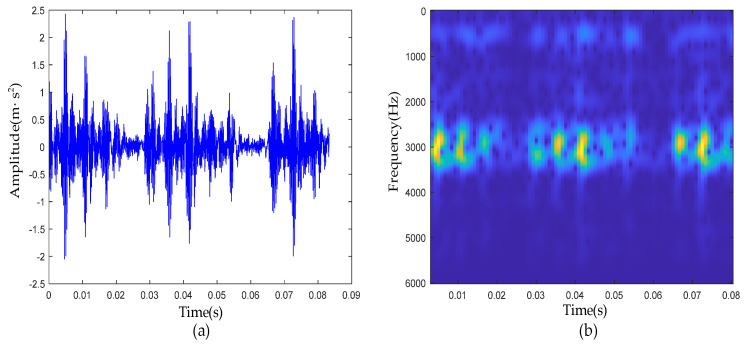
The signal of inner-race defective bearing: (**a**) waveform of raw signal; (**b**) time-frequency diagram of raw signal.

From the waveform of the raw vibration signal (Figure 6a) and the time-frequency analysis result (Figure 6b), periodic impulses were corrupted by strong noise.

**Figure 7:**
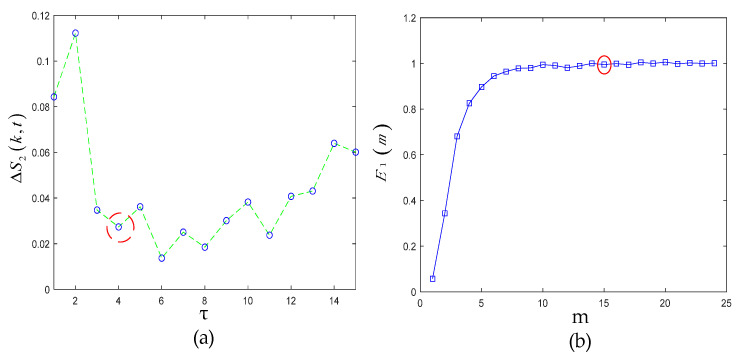
(**a**) $\Delta S_{2}\left(k,t\right)$ curve; (**b**) E1 curve.

First, the C-C method and Cao’s method were used to jointly determine the embedded dimension and time delay in PSR. The change curve of $\Delta S_{2}\left(k,t\right)$ with τ in the C-C method is shown in Figure 7a. It can be clearly seen from the red circle in the figure that when τ was 4, $\Delta S_{2}\left(k,t\right)$ took the first local minimum value. Therefore, it can be determined that the optimal solution of τ should be 4. 

Next, taking τ=4 as the input of Cao’s method, the curve of E1 was shown, as in Figure 7b. It can be seen when m was greater than 14, the value of E1 remained basically unchanged, so the final value of the phase space embedding dimension m of the measured signal was 14.

After solving the time delay and embedded dimension of the defective signal by the C-C method and Cao’s method, fuzzy entropy and the mean-square error (MSE) were used in this study as the quantitative evaluation index of the PSR to characterize the complexity and distortion of the reconstructed space. A larger fuzzy entropy reflected that the high-dimensional space obtained by PSR was more complex and the intrinsic structure was more fuzzy. A smaller fuzzy entropy reflected that the high-dimensional space had less complexity and the signal had higher SNR. MSE represents the difference between the reconstructed space of the PSR and the raw signal. A greater MSE means a more serious the degree of signal distortion. The specific calculation results are shown in Table 2.

Compared with the other two algorithms, the combined algorithm of C-C and Cao had the smallest fuzzy entropy and the smallest MSE, which means the high dimension space obtained by PSR had low complexity and distortion.

#### 4.1.2. Experimental Results of ETFM

**Figure 8:**
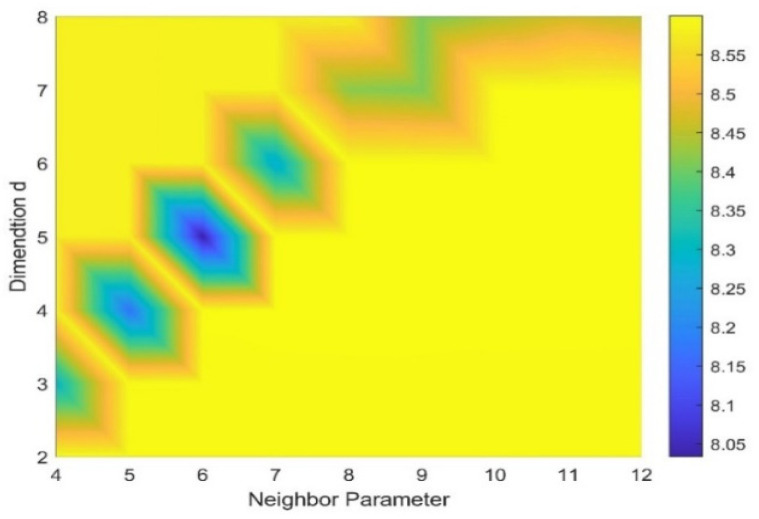
Gridded parametric search method results.

After PSR, we chose the time-frequency manifold learning algorithm LLTSA to further mine and de-noise the high-dimensional space of the signal. The Renyi entropy value of TFM obtained by LLTSA was calculated by using the gridded parameter search method. The specific results are shown in Figure 8. It can be clearly seen that when d = 5 and k = 6, the ETFM with the smallest Renyi entropy was obtained.

**Figure 9:**
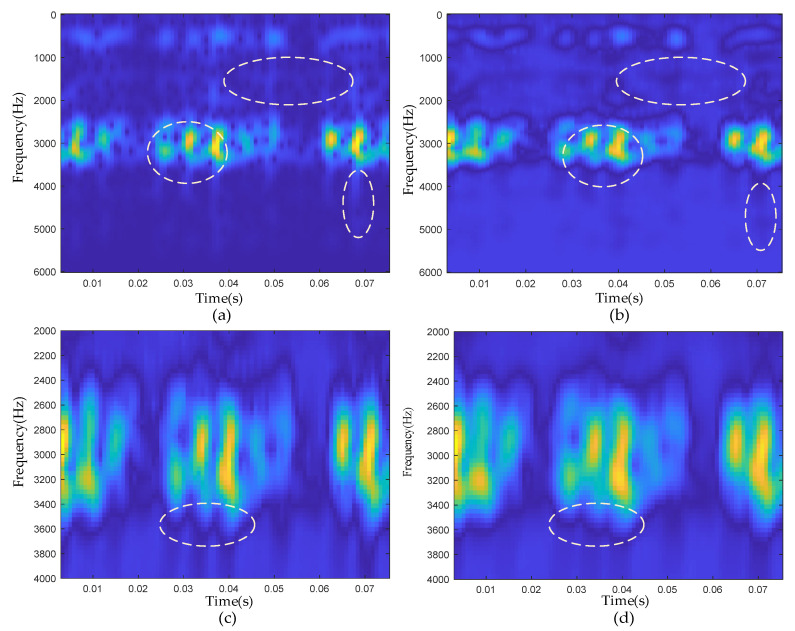
Time-frequency analysis results: (**a**) raw signal time-frequency diagram; (**b**) ETFM signature; (**c**) partial enlargement of ETFM; (**d**) partial enlargement of LTSA-TFM.

The comparison between the time-frequency diagram of the raw signal and the ETFM is shown in Figure 9a,b. As marked in the figure, the low-dimensional manifold time-frequency diagram was completed to retain the transient pulses of the raw signal in the 3000 Hz resonance band, and there was also a certain noise suppression effect in the resonance band and other frequency bands. As seen from Table 3, compared with the raw signal, the Renyi entropy value of ETFM was much smaller, which proves that the noise suppression effect of the time-frequency manifold algorithm was powerful. Compared with the Renyi entropy of LLTSA-TFM and LTSA-TFM without optimization, ETFM also had advantages, which proved the effectiveness of the parameter optimization method on the basis of grid search. Figure 9c,d, shows the enlarged comparison of the ETFM and the LTSA-TFM near the resonance band. Both algorithms can effectively retain pulses in the resonance band of 3000 Hz. However, from the places marked in the figure, it can be clearly seen that the pulses in ETFM were more independent and obvious than that in LTSA-TFM.

#### 4.1.3. Experimental Results of Signal De-Noising

**Figure 10:**
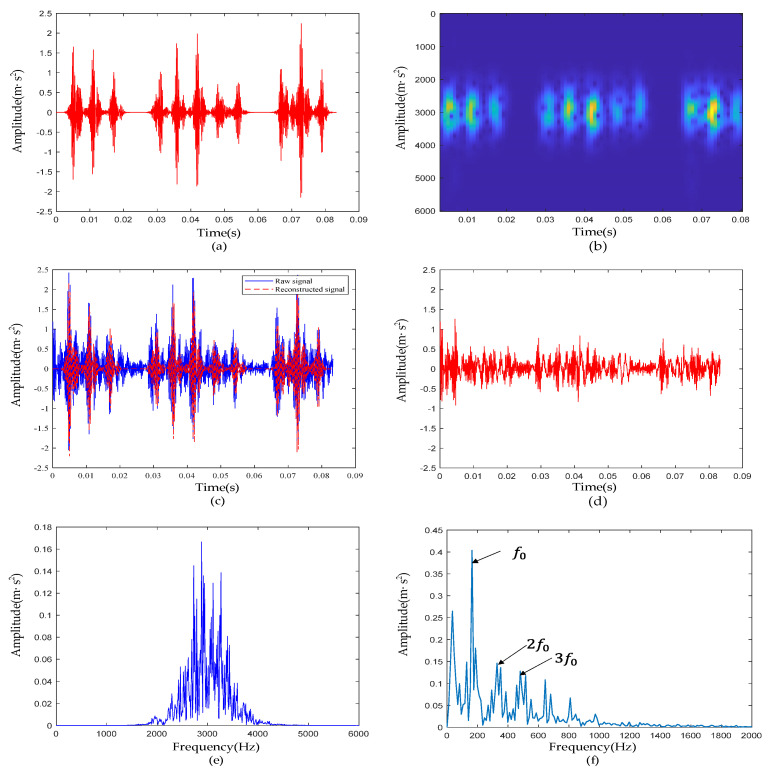
The reconstruction results of the inner-race defective bearing signal: (**a**) waveform of the reconstructed vibration signal; (**b**) time-frequency diagram of the reconstructed signal; (**c**) comparison of the reconstructed signal and raw signal; (**d**) waveform of the residual signal; (**e**) reconstructed signal spectrum; (**f**) reconstructed signal envelope spectrum.

The complete framework of the method proposed in this paper was used to reconstruct the inner-race defective signal, and its time domain waveform is shown in Figure 10a. By comparing the raw signal with the reconstructed signal (Figure 10c) and the residual signal (Figure 10d), we can see that the reconstructed signal accurately reproduced the transient pulses in the raw signal, and the noise between transient pulses was largely suppressed. From Figure 10b,e, the noise outside the resonance band at approximately 3000 Hz was basically filtered out, and the extracted transients of interest were represented with a certain noise suppression effect. According to Table 1, the characteristic frequency $f_{0}$ of the inner-race defective signal was 162 Hz. In Figure 10f, $f_{0}$ and its harmonics $\left(2f_{0},3f_{0}\right)$ are clearly visible, and they were not disturbed by harmonics and noise, which proves the ability of the proposed method to reveal fault characteristics.

### 4.2. Experimental Results of Outer-Race Defective Signal

**Figure 11:**
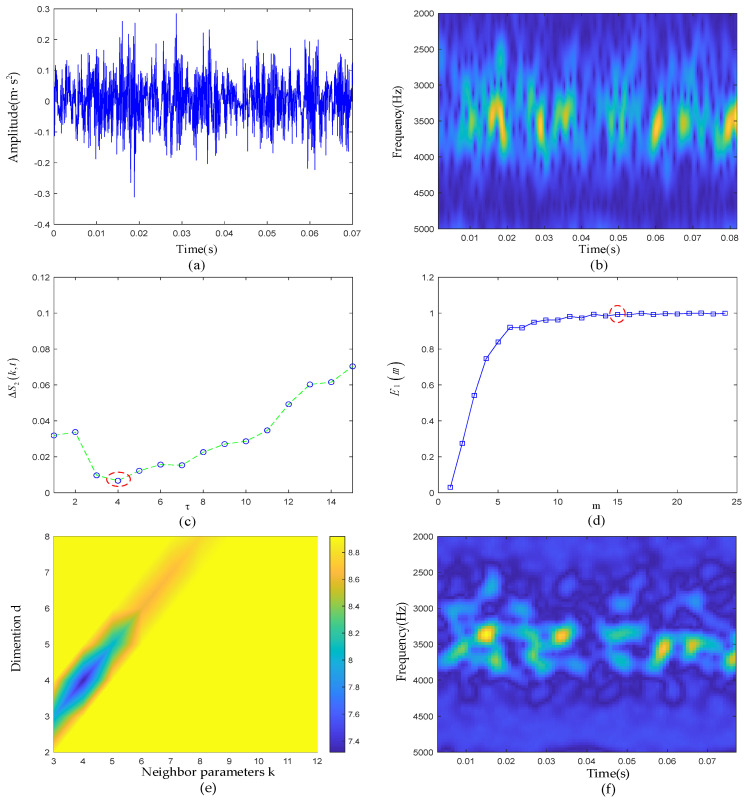
(**a**) Waveform of the raw signal of the outer ring fault; (**b**) time-frequency diagram of the raw signal; (**c**) $\Delta S_{2}\left(k,t\right)$ curve; (**d**) E1 curve; (**e**) gridded parametric search method results; (**f**) ETFM signature.

In this subsection, we analyze the outer-race defective signal. Due to the existence of strong background noise, no obvious defective transient characteristics could be seen on the raw signal time-domain waveform and time-frequency diagram. First, the parameters of PSR were determined jointly by the C-C method and Cao’s methods. As shown in Figure 11c,d, it can be clearly seen that when τ was set to 4, $\Delta S_{2}\left(k,t\right)$ took the first minimum value. Then, taking τ as 4 as the input of Cao’s method, the relationship between the *E*1 value and m was obtained. When *m* was greater than 15, the *E*1 value remained basically unchanged. Therefore, the phase space embedding dimension *m* of the measured signal finally took the value of 15. In the time-frequency manifold reconstruction, the input parameters of the LLTSA algorithm were determined according to the grid search method. The specific results are shown in Figure 11e. When *d* = 4, and *k* = 4, the Renyi entropy was the smallest. The time-frequency diagram of the ETFM is shown in Figure 11f. Compared with Figure 11b, it can be seen that the manifold learning algorithm had a certain degree of noise suppression effect, and the transient pulses in the time-frequency diagram was clearer, highlighting the defective characteristics.

**Figure 12:**
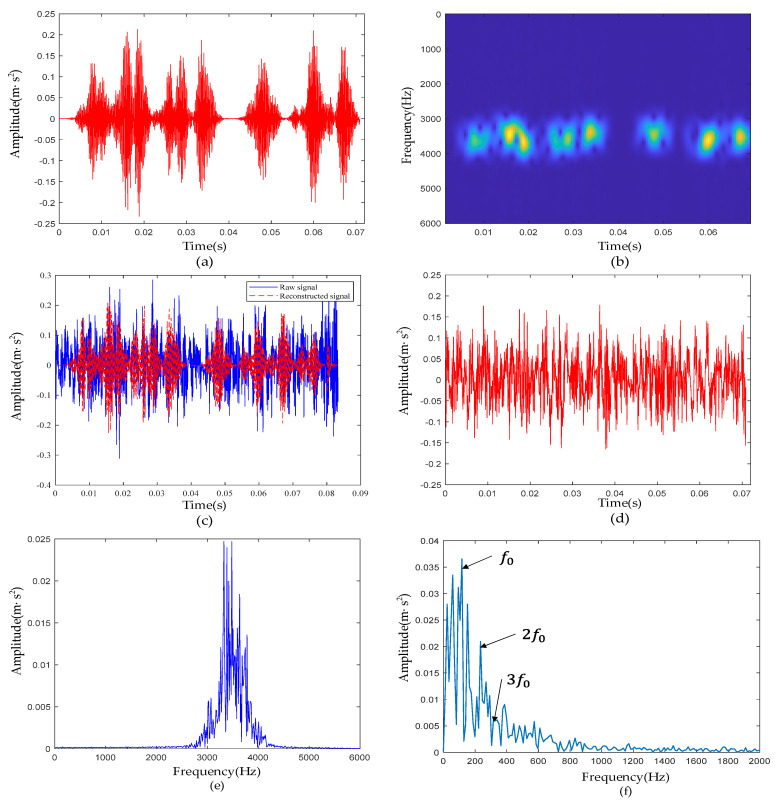
Outer-race defective signal de-nosing results: (**a**) Waveform of reconstructed signal; (**b**) reconstructed signal time-frequency diagram; (**c**) comparison of reconstructed signal and raw signal; (**d**) waveform of the residual signal; (**e**) reconstructed signal spectrum; (**f**) reconstructed signal envelope spectrum.

Figure 12a is the waveform of the reconstructed signal. Figure 12b is the time-frequency diagram of the reconstructed signal. Compared with Figure 11b, the noise outside the resonance band was essentially filtered out, and the impulses between 3000 Hz and 4000 Hz were reconstructed. From the comparison of the reconstructed signal and the raw signal and the residuals of the two, it can be seen that most of the noise between the impulses did not appear in the reconstructed signal. According to Table 1, the characteristic frequency $f_{0}$ of the outer-race defective signal was 105 Hz. In Figure 12f, $f_{0}$ and its harmonic $2f_{0}$ are clearly visible; $3f_{0}$ was not clear and was affected by harmonics and noise.

### 4.3. Experimental Results of Rolling-Element Defective Signal

**Figure 13:**
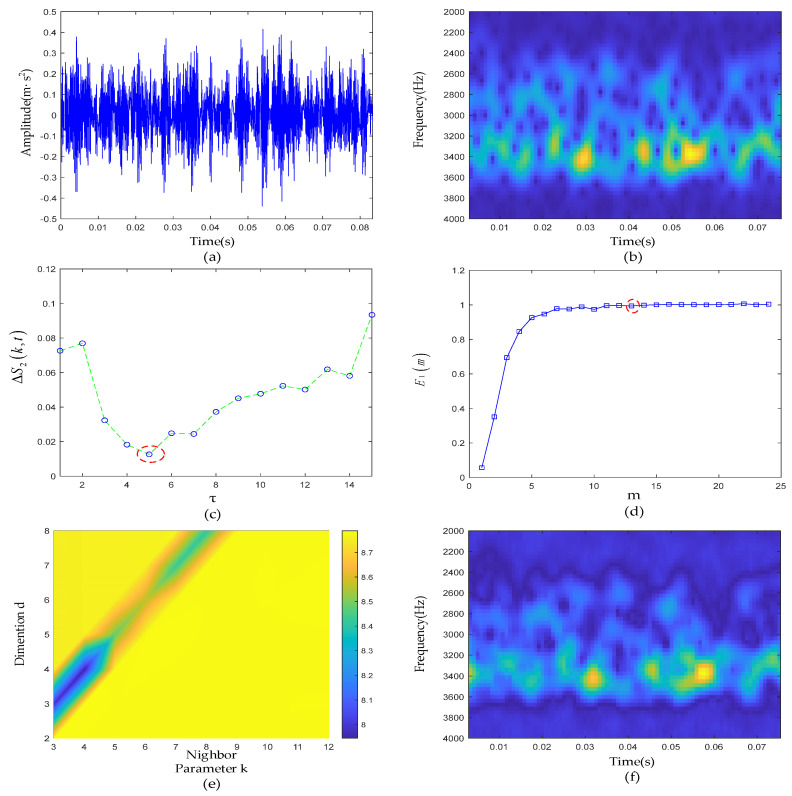
Rolling-element defective signal de-nosing results: (**a**) Waveform of the raw signal of the rolling-element fault; (**b**) time-frequency diagram of the raw signal; (**c**) $\Delta S_{2}\left(k,t\right)$ curve; (**d**) E1 curve; (**e**) gridded parametric search method results; (**f**) ETFM signature.

**Figure 14:**
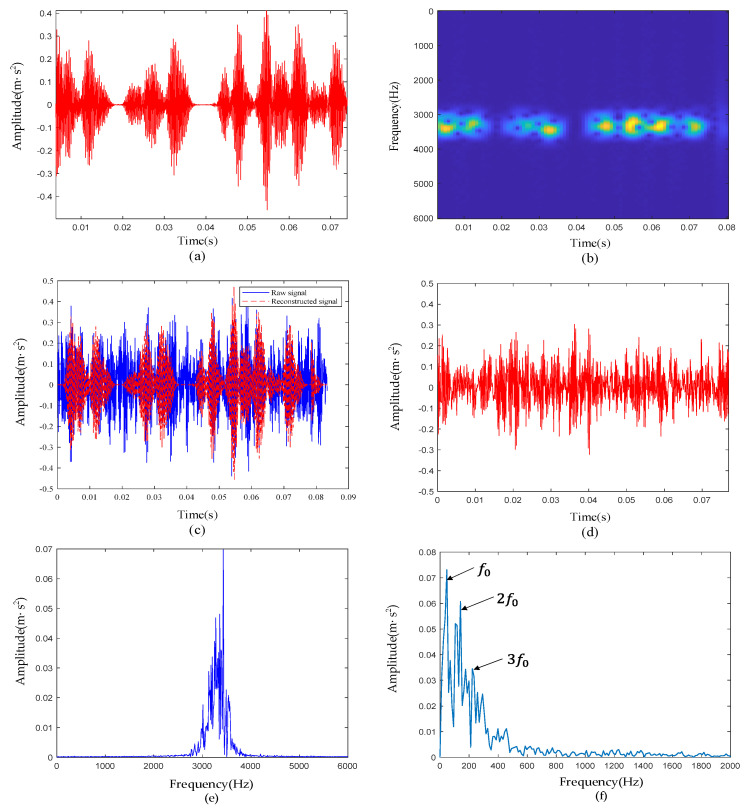
(**a**) Waveform of reconstructed signal; (**b**) reconstructed signal time-frequency diagram; (**c**) comparison of reconstructed signal and raw signal; (**d**) waveform of the residual signal; (**e**) reconstructed signal spectrum; (**f**) reconstructed signal envelope spectrum.

In this subsection, the rolling-element defective signal is analyzed. The reconstruction analysis results are shown in Figure 13. The raw signal waveform is shown in Figure 13a, and the time-frequency analysis results are shown in Figure 13b. Noise was distributed over the entire time-frequency domain. First, the parameters of the PSR were jointly determined by the C-C method and Cao’s methods. As shown in Figure 13c,d, it can be clearly seen that when τ was set to 5, $\Delta S_{2}\left(k,t\right)$ took the first local minimum, so it can be determined that the optimal solution of τ should be 5 in the parameter selection of PSR. Then, taking τ as 5 as the input of Cao’s method, the relationship between the *E*1 value and m was obtained. When m was greater than 13, the *E*1 value essentially remained unchanged. Therefore, the phase space embedding dimension m of the measured signal finally took the value of 13. The final result of the same grid search method is shown in Figure 13e. When *d* = 3, and *k* = 3, the Renyi entropy was the smallest. The time-frequency diagram of the ETFM is shown in Figure 13f. The sparsity of the OMP algorithm was set to 8, and the reconstruction results are shown in Figure 14a,b. It can be clearly seen that the reconstructed signal accurately reproduced all the pulses in the raw signal and had an certain noise suppression effect outside the resonance band noise. The noise between transient pulses in the resonance band was also filtered to some extent by the effect. According to Table 1, the characteristic frequency $f_{0}$ of the rolling-element defective signal was 60 Hz. In Figure 14f, $f_{0}$ and its harmonics $\left(2f_{0},3f_{0}\right)$ are clearly visible and were not disturbed by harmonics and noise, which proves the ability of the proposed method to reveal fault characteristics.

## 5. Discussion

### 5.1. Ablation Experiment

**Figure 15:**
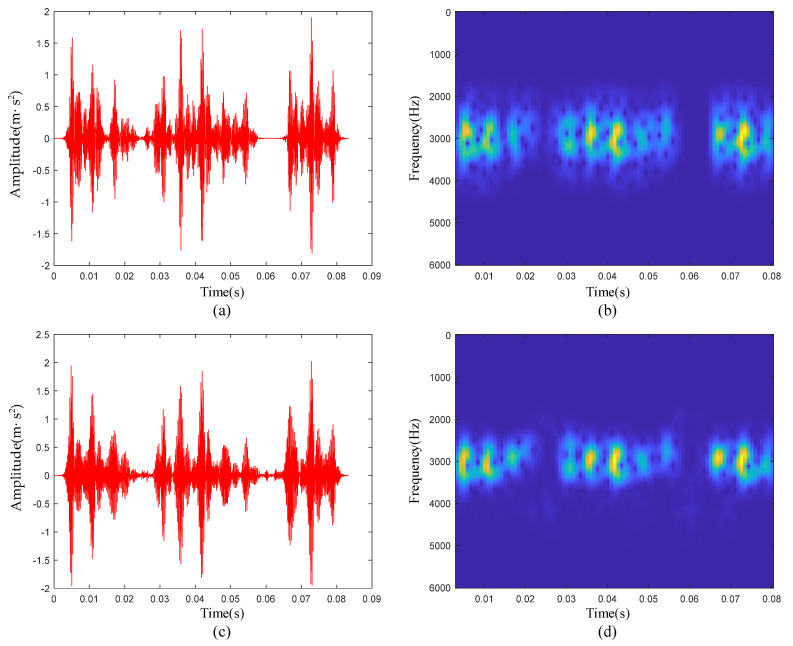
The reconstruction results obtained by method 1 and method 2: (**a**) waveform of the reconstructed signal by method 1; (**b**) time-frequency diagram of the reconstructed signal by method 1; (**c**) waveform of the reconstructed signal by method 2; (**d**) time-frequency diagram of the reconstructed signal by method 2.

In order to prove the effectiveness of our innovative work on ETFM and kurtosis-wavelet dictionary, an ablation experiment is necessary. First, we did not use the ETFM to train the dictionary but used the raw signal time-frequency map while keeping the rest of the method framework the same, which is referred to as method 1 below. From the time-domain waveform (Figure 15a) and time-frequency diagram (Figure 15b) of the final reconstructed signal, it can be seen that method 1 still had a certain noise suppression effect. However, compared with Figure 10a,b, Figure 15a,b shows more noise between extracted transients, but the defective feature extraction effect was general, which also verifies the de-noising effect of the TFM to a certain extent.

Then, we no longer used the kurtosis-wavelet dictionary, but set time-frequency atoms to form a dictionary in the entire signal time domain, which is referred to as method 2 below. The waveform is shown in Figure 15c. It can be clearly seen that method 2 also reconstructed the transient pulses in the raw signal, and the noise suppression effect was stronger than the result obtained by method 1, but it was still not as good as the result in Figure 10a,b. A lot of noise appeared, which further proves that the kurtosis-wavelet dictionary can not only reconstruct the transients of interest but also have a strong de-noising effect. 

Table 4 lists the average residual between the reconstructed signal and the raw signal, the energy of the reconstructed signal, and Renyi entropy. The reconstructed signal obtained by the method proposed in this paper had the smallest Renyi entropy value, the highest instantaneous frequency aggregation degree, and the best noise suppression effect. However, the average residual was the largest and the signal energy was the smallest, because method 1 and method 2 introduced noise in the reconstruction process, making the value of MSE smaller. On the basis of the above analysis, the innovative work in the ETFM and kurtosis-wavelet dictionary in this paper plays an important role in the noise suppression effect of the reconstructed signal. However, there still are some limitations.

The time-frequency matrix obtained by the STFT was too large, and the size increased with the length of the signal. This would greatly increase the computational cost of ETFM and kurtosis-wavelet dictionary generation. In the process of dictionary construction, after calculating the kurtosis of each segment of the signal, the number of signal segments we chose to set atoms in this position was generally determined by the raw signals, and automatic selection was unable to be achieved. These issues need to be further addressed in our future work.

### 5.2. Comparison with Other Methods

**Figure 16:**
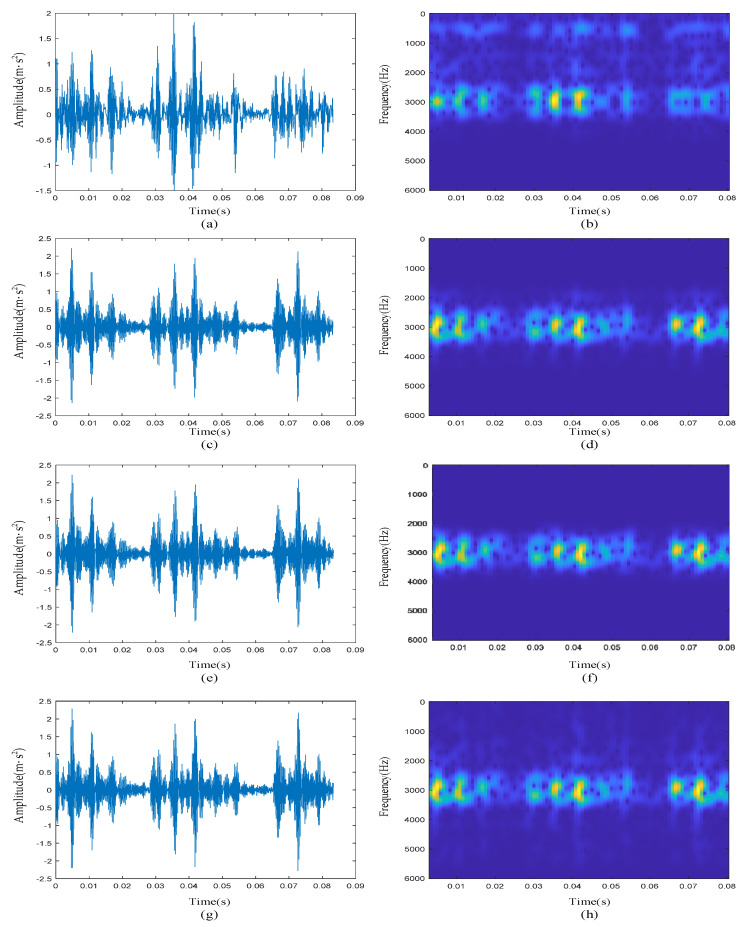
The reconstruction results obtained by other methods: (**a**) waveform of reconstructed signal obtained by DWT; (**b**) time-frequency diagram of reconstructed signal obtained by DWT; (**c**) waveform of reconstructed signal obtained by CWT; (**d**) time-frequency diagram of reconstructed signal obtained by CWT; (**e**) waveform of reconstructed signal obtained by WPT; (**f**) time-frequency diagram of reconstructed signal obtained by WPT; (**g**) waveform of reconstructed signal obtained by EMD; (**h**) time-frequency diagram of reconstructed signal obtained by EMD.

To compare the signal de-noising effect of the method proposed in this paper, four traditional filtering algorithms were used, and Figure 16 shows the final reconstruction results.

1. Discrete wavelet transform (DWT): the specific reconstruction effect is shown in Figure 16a,b. It can be clearly seen from the time-frequency diagram that the high-frequency noise above 4000 Hz was basically filtered out, but the noise suppression effect in other frequency bands was poor. Compared with the time-frequency diagram of the raw signal, the transient pulses at approximately 0.07 s were not reconstructed, resulting in a certain degree of loss of defective features.
2. Continuous wavelet transform (CWT): the specific reconstruction effect is shown in Figure 16c,d. Compared with the reconstruction result in Figure 16a,b, it had a good noise suppression effect in both the low-frequency band and high-frequency band, and completely reconstructed all transient pulses. However, the signal de-noising effect was poor at the resonance frequency of 3000 Hz.
3. Wavelet packet transform (WPT): the specific reconstruction effect is shown in Figure 16e,f. The number of decomposition layers of WPT was 3. Since the sampling frequency of the signal was 12 kHz, according to Nyquist’s law, the frequency difference of the nodes in the third-level wavelet tree was 6000/8 = 750 Hz. Here, we chose the fourth and fifth wavelet nodes whose frequencies ranged from 2250 to 3750 Hz. From the time-frequency analysis results of the reconstructed signal, it can be clearly seen that compared with the filtering algorithm of CWT, the frequency band at the center frequency of 3000 Hz was narrower. However, the noise in the resonance band was still not filtered out, and it can be seen from Figure 16e that the transient pulses were submerged in a large amount of noise.
4. The filtering effect based on the EMD algorithm is shown in Figure 16g,h, and the IMF whose center frequency was approximately 3000 Hz was selected as the reconstructed signal. It can be seen from its time-frequency diagram that the reconstructed signal contained considerable noise because the EMD algorithm had bandpass filtering characteristics for white noise.

**Figure 17:**
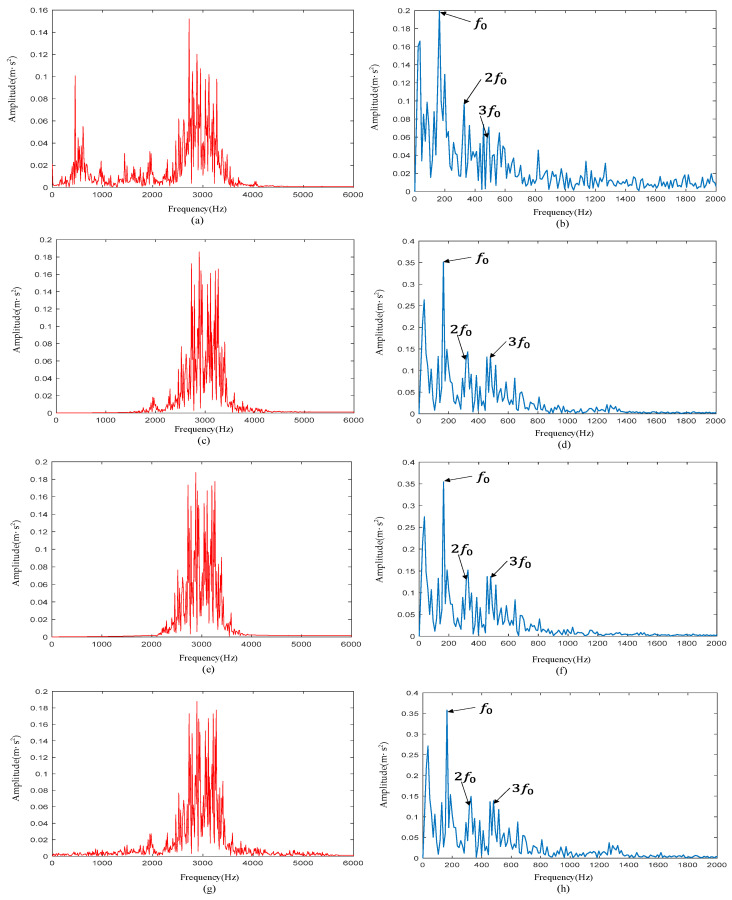
(**a**) Signal spectrum obtained by DWT; (**b**) signal envelope spectrum obtained by DWT; (**c**) signal spectrum obtained by CWT; (**d**) signal envelope spectrum obtained by CWT; (**e**) signal spectrum obtained by WPT; (**f**) signal envelope spectrum obtained by WPT; (**g**) signal spectrum obtained by EMD; (**h**) signal envelope spectrum obtained by EMD.

According to Table 1, the characteristic frequency $f_{0}$ of the inner-race defective signal was 162 Hz. As shown in Figure 17, there was a large amount of noise in the envelope spectrum of reconstructed signals obtained by other methods. $2f_{0}\;\mathrm{and}\;3f_{0}$ were drowned in the noise and were difficult to distinguish. Comparing the analysis results in Figure 10f, we can see the effectiveness of the proposed method in extracting bearing fault features.

Table 5 shows the comparison between the above four filtering algorithms and the method proposed in this paper. It can be clearly seen that the Renyi entropy of the time-frequency diagram of the final reconstructed signal obtained by the method proposed in this paper was the smallest among all algorithms, and the kurtosis of the reconstructed signal was nearly twice that of other algorithms. Combining the results in Figure 16 and Table 5, it can be proven that compared with other methods, the structure of the method proposed in this paper can not only accurately reconstruct the transient pulses in the raw signal, but also make the fault characteristics more prominent and has an excellent signal de-noising effect in each frequency band.

## 6. Conclusions

In this paper, a new signal de-noising method is proposed that can improve the SNR and identify the weak pulses generated by the early faults of rolling bearings. In the phase space reconstruction, we propose that the C-C method and Cao’s method jointly determine the optimal time delay and embedding dimension, which reduces the distortion of the reconstructed space and excavates the high-dimensional characteristics of the signal. The proposed enhanced time-frequency manifold improves the noise suppression effect of manifold learning to a certain extent. Compared with other methods, ETFM has better time-frequency aggregation and more prominent fault pulse. The dictionary trained by ETFM has a better matching degree with the transient pulses in the signal. The proposed kurtosis-wavelet dictionary can reduce the number of atoms matching the noise in the raw signal. The reconstructed signal obtained by sparse representation not only retains the amplitude and phase information of the raw signal but also has a better noise suppression effect, and the impact pulses of the reconstructed signal are more prominent. Compared with other filtering algorithms, the kurtosis of reconstructed signal obtained by the denoising method proposed in this paper was found to be 12.266, nearly twice that obtained by other methods. The Renyi entropy value of 7.648 was the lowest among all methods, and it can be seen from the time-frequency diagram that the reconstructed signal had a better noise suppression effect in all frequency bands. In addition, the algorithm proposed in this paper had good results in the application of defective signals of inner-race, outer-race, and rolling-element. This signal de-noising method has a certain application value for early fault diagnosis and defect feature extraction of bearings. However, the proposed method still has some problems, such as a too long computation time, and the time-frequency resolution is limited by the performance of the algorithms. In future research, we will optimize the computational speed of the used algorithms and apply higher precision time-frequency analysis algorithms to identify the noise in the signal.

## Figures and Tables

**Table 1:** Parameters of the faulty bearing.

Defect Type	Inner-Race	Outer-Race	Rolling Element
Defect size	0.5334 mm	0.3556 mm	0.1778 mm
RPM	1797	1750	1750
Characteristic Frequency	162 Hz	105 Hz	60 Hz

**Table 2:** Comparing parameters of phase space.

Control Parameters	C-C	Cao	C-C + Cao
Fuzzy entropy	0.2641	0.2657	0.2619
MSE	0.2494	1.0324	0.2479

**Table 3:** Renyi entropy of TFM.

Control Parameters	Raw Signal	ETFM	LLTSA-TFM	LTSA-TFM
Renyi entropy	8.211	8.033	8.192	8.201

**Table 4:** Comparison of reconstructed signal parameters.

Methods	MSE	Reconstructed Signal Energy	Reconstructed Signal Renyi Entropy
The proposed method	0.0214	12.267	14.476
Method 1	0.0213	12.471	15.666
Method 2	0.0213	13.845	15.750

**Table 5:** Comparison of other methods.

Algorithms	Renyi Entropy	Kurtosis of the Reconstructed Signal
The proposed method	7.648	12.266
DWT	8.283	7.307
CWT	7.697	6.350
WPT	8.227	5.419
EMD	7.931	6.464

## Data Availability

Not applicable.
